# Molecular docking analysis of marine compounds with voltage gated calcium channel for potential anti-epileptic molecules

**DOI:** 10.6026/973206300200271

**Published:** 2024-03-31

**Authors:** Deepak Sheokand, Annu Grewal, Pawan Kumar, Raveena Chauhan, Vandana Saini, Ajit Kumar

**Affiliations:** 1 Toxicology and Computational Biology Group, Centre for Bioinformatics, Maharshi Dayanand University, Rohtak, Haryana, India, 124001

**Keywords:** Molecular docking, Seizure, Voltage-gated calcium channel

## Abstract

Epileptic seizures are directly linked with an anomalous influx of extracellular calcium or sodium anions through voltage-gated channels disturb the chemical and electrical gradients, resulting in seizures or jerking moments. Voltage-gated calcium channel (VGCC) subunit α2δ-1 is the binding site for gabapentinoids used to treat epilepsy and neuropathic pain. However, this class of drugs showed severe side effects associated with CNS and respiratory depression. Hence, we screened a total of 2583 phytochemicals from the Comprehensive Marine Natural Products Database for their drug likeliness and pharmacokinetics (ADME/T) properties. The selected phytochemicals were docked with the VGCC α2δ-1 protein target and the marketed AED Pregabalin is used as standard. The docking results helped to select 45 docked compounds with better binding affinity, among which Acanthiline A showed the maximum binding affinity with the binding energy of -11.9 kcal/mol, thus reflecting its potential anti-epileptic activity.

## Background:

Epilepsy is a chronic disease characterised by two or more recurrent seizures [1]. Other epileptic symptoms include unconditional body movement, loss of consciousness, and associated psychological conditions, including anxiety and depression. There are several reasons known to cause epilepsy, including an imbalance of nerve-signalling chemicals or ions, tumours, strokes, brain damage from illness or injury, or a combination of these factors [2]. Genetic factors or mutations in associated ion channels have also been reported to be the leading cause of epilepsy. Hence, most anti-epileptic drugs (AEDs) target the inhibition of ion channels. Voltage-gated sodium channel (VGSC) inhibitors are first-line epileptic therapeutics, followed by voltage-gated calcium channel (VGCC) blockers [3]. VGCC α2δ-1 also carries a motif MIDAS (297-301) which is present in VWFA Domain, binds to divalent metal cations and is required to promote trafficking of the α subunit to the plasma membrane [4,5]. There are multiple cysteines in both α2 and δ making it look like there are both intra and inter subunit disulphide bonds. The process of disulphide linking and proteolytic cleavage of α2 and δ must occur during trafficking of α2 protein. Cellular localisation of α2δ subunit: α2δ-1, α2δ-2, α2δ-3, α2δ-4 in which the α2δ-1 is found to be widely distributed throughout the brain at both m-RNA and protein level which is involved in cortical processing, learning and memory, defensive behaviour, neuroendocrine secretion and, primary sensory transmission [4]. α2δ subunit proteins can be further enhanced by the presence of multiple splice variants. These are created by alternative splicing. α2δ splice variant A/B (containing both regions) is only present in lung and skeletal muscle but is not present in brain tissue.

The α2δ-1 subunit of VGCC regulates calcium current density for the activation-inactivation kinetics of the calcium channel, which is molecularly characterised by the binding of gabapentinoids (gabapentin (GBP) and pregabalin) [6]. Gabapentinoids have been shown to inhibit the release of a wide range of neurotransmitters. α2δ splice variant B/C and C both bind gabapentin but C variant binds drug with 10 times reduced affinity (C. Dolphin 2012). In α2δ mutant mice, studies showed no binding of drugs pregabalin and GBP; hence, these mice lack therapeutic analgesic and anticonvulsant activity [7]. Pregabalin (Lyrica) is a second-generation AED, which is 2-10 times more potent than gabapentin and is approved for managing the neuropathic pain associated with epilepsy [8]. Also, pregabalin is significantly effective in achieving more than a 50% reduction in seizures while treating partial epilepsy patients [9]. However, their use is often limited by adverse effects like CNS and respiratory depression, and incomplete seizure control, underscoring the need for alternative agents with improved efficacy and safety profiles. Marine plants produce a diverse array of bioactive compounds that have shown promise for the management of neurological disorders, including epilepsy [10, 11]. Several studies have reported the anticonvulsant effects of extracts and isolated compounds from marine macroalgae. For instance, fucosterol isolated from the brown algae Sargassum fusiforme displayed anticonvulsant effects in mice [12]. Extracts of the brown algae Sargassum ilicifolium and Ecklonia cava also exhibited anticonvulsant activities in vivo [13, 14]. Furthermore, an anticonvulsant compound was isolated from the marine diatom Skeletonema marinoi in a bioassay-guided fractionation study [15]. Therefore, it is of interest to document the molecular docking analysis of voltage gated calcium channel with marine compounds for screening potential anti-epileptic molecules in drug discovery.

## Materials & Methods:

## Retrieval of phytochemicals and Target receptor selection:

The Comprehensive Marine Natural Products Database (CMNPD) is a unique resource that compiles extensive information on natural products derived from marine organisms. The CMNPD aims to serve as a knowledge base that collates and organizes the substantial research output on marine natural products, providing a centralized platform to facilitate further discovery and development in this domain. The total 2583 marine phytochemicals along with physiochemical properties like molecular weight, logP, H bond acceptor etc was downloaded from CMNPD in csv format. VGCC subtype α2δ-1 was searched over UniProt for protein study with UniProt ID: P54289, and its tertiary structure was retrieved from the RCSB-PDB database with PDB ID: 7VFS. Only chain-B is the tertiary structure of protein; hence all non-standard amino acids and other chains except chain-B were deleted using UCSF Chimera. The remaining chain-B was energy minimised using 100 steps of the steepest descendant algorithm under AMBER ff14sb force field. The energy-minimised protein file was saved in pdf file format.

## Drug likeliness and Pharmacokinetics screening:

The initial screening process involved evaluating the drug-likeness properties of the 2583 compounds retrieved from the database. This was accomplished by applying Lipinski's rule of five, a widely-used set of guidelines that help identify compounds with favorable pharmacokinetic properties. The phytochemicals that satisfied the drug-likeness criteria were subsequently subjected to a comprehensive evaluation of their absorption, distribution, metabolism, excretion, and toxicity (ADME/T) properties using the ADMETlab2.0 web server. This step aimed to identify compounds with favorable pharmacokinetic profiles and minimal toxicity concerns. Selected phytochemicals were checked for their absorption (Human intestinal absorption and oral bioavailability), BBB permeability and toxicity (hepatotoxicity, Ames's mutagens, carcinogens, and toxicophores). Phytochemicals exhibiting favorable absorption-related properties, BBB permeability, and negative scores for toxicity parameters were prioritized for subsequent molecular docking studies.

## Phytochemical & standard drugs structure preparation for docking:

Promising AED pregabalin is already in use for targeting VGCC subtype α2δ; hence, was selected as standard drug. 3D structural file for standard drug pregabalin was retrieved in sdf format from Drugbank with the accession number DB00230. Whereas 3D structure of phytochemicals with favourable pharmokinetics properties were retrieved CMNPD. All the 3D structures of selected phytochemicals as well as standard drug were dock prep using UCSF-Chimera and were saved as pdb files.

## Molecular docking studies:

Auto dock vina was used to virtually screen selected phytochemiclas against epileptic receptor VGCC α2δ-1. PyRx tool was used to compound conversion in pdbqt format and to automate docking study. AEDs binding region on receptor α2δ was selected as the binding pocket for our study, and a grid box was generated with grid parameters (dimensions (33.7506, 46.3849, 34.3605), and center (185.1339, 226.8722, 145.6644)) was generated with the spacing of 1 Å. For each dock, 100 conformations were generated. The top three docked ligands with the highest binding affinities were selected for further analysis using PyMOL v2.4.0. The binding conformations were visually inspected, with emphasis on the poses exhibiting the maximum number of binding clusters at the receptor's binding site, as these represent the most favourable binding orientations. The polar and electrostatic interactions within the ligand-receptor complexes were analyzed using LigPlot+ v2.2, enabling the identification of the specific amino acid residues involved in the interactions.

## Results & Discussion:

A library of 2583 compounds, retrieved from the Comprehensive Marine Natural Products Database (CMNPD), underwent a preliminary assessment of their drug-likeness properties using Lipinski's rule of five. This rule provides guidelines for evaluating the oral bioavailability of small molecules based on their physicochemical parameters. This filter retained 1485 compounds exhibiting favourable drug-like characteristics. The selected compounds were further evaluated for their absorption, distribution, metabolism, excretion, and toxicity (ADME/T) profiles using the ADMETlab 2.0 web server. This platform employs machine learning algorithms trained on extensive experimental data to predict various ADME/T properties. Blood-brain barrier (BBB) permeability screening identified 677 phytochemicals as potential BBB-permeable candidates, a crucial criterion considering the target receptor association with neurological disorders. These 677 BBB-permeable compounds were further prioritized based on their predicted pharmacokinetic properties, including oral bioavailability, and toxicity profiles. An optimal probability cutoff range of 0 to 0.7 was applied to select compounds with high gastrointestinal absorption, and BBB permeable properties. Ultimately, our computational screening workflow yielded a focused subset of 150 phytochemicals exhibiting drug-like properties, BBB permeability, favourable absorption profiles (Human intestinal absorption and oral bioavailability), and minimal predicted toxicity (hepatotoxicity, Ames's mutagens, carcinogens, and toxicophores), making them promising candidates for subsequent molecular docking studies against the target receptor.

**Figure 1:**
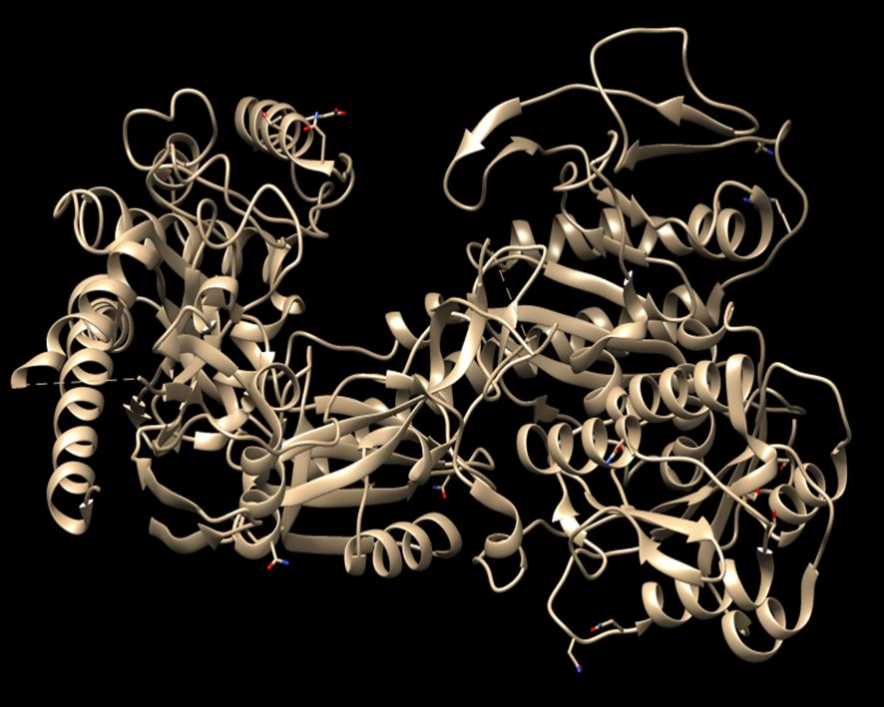
3D structure of VGCC α2δ-1 subunit selected for molecular docking studies

**Figure 2:**
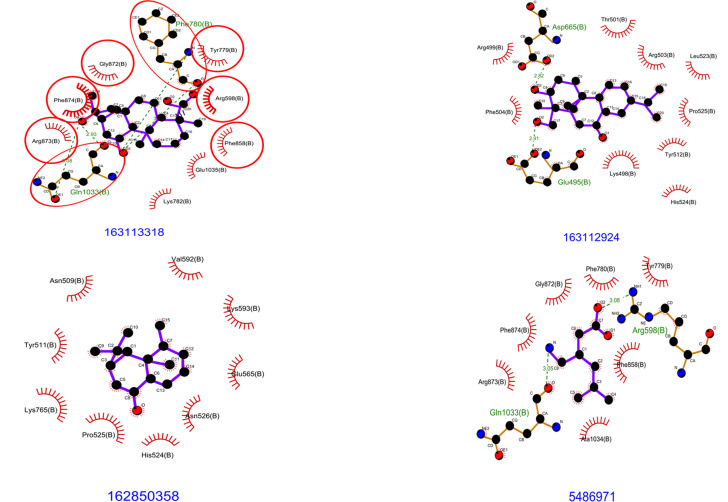
The docking site images of VGCC α2δ-1 protein when docked against ligands CMNPD31502 (163113318), CMNPD31516 (163112924), CMNPD23679 (162850358) and Pregabalin (5486971)

Molecular docking simulations using Auto dock vina were performed to evaluate the binding interactions of the selected 150 marine phytochemicals with the human, voltage-gated calcium channel (VGCC) α2δ-1 subunit, a key therapeutic target for antiepileptic activity. The crystal structure of VGCC α2δ-1 subunit (PDB ID: 7VFS) was used as the receptor for docking studies (Figure 1), and pregabalin, a marketed VGCC inhibitor, was employed as the standard reference compound. Pregabalin is clinically used anticonvulsants that target the α2δ-1 subunit, leading to the inhibition of calcium influx into neurons and subsequent therapeutic effects [7,8].Pregabalin demonstrated a binding energy of -6.8 Kcal/mol (Table 1). Notably, the 45 identified phytochemicals exhibited more favorable binding energies ranging from -11.9 to -6.9 kcal/mol (Table 1). Among the top hits, CMNPD31502 (Acanthiline A from Acanthus ilicifolius) demonstrated the highest binding affinity of -11.9 kcal/mol, followed by CMNPD31516 (Dencandrol f from Ceriops decandra) with a binding energy of -10.9 kcal/mol. It is noteworthy that several other phytochemicals, such as CMNPD23679 (ent-1(10)-aristolen-9β-ol from Laurencia similis) and CMNPD29266, also exhibited binding energies below -8.9 kcal/mol, indicating their potential as promising lead compounds for further investigation. Acanthiline A and the standard drug pregabalin share a common hydrogen bond interaction with the Gln1033 residue (Table 2 & Figure 1). Additionally, Acanthiline A shraes common interacting residue like Arg598, Tyr779, Phe780 ,Lys782, Phe858,Gly872, Arg873, Phe874, with residue involved in pregabalin binding (Table 2 & Figure 2). These similarities suggest that Acanthiline A occupy a similar binding pocket as pregabalin, potentially contributing to its high binding affinity and suggesting its potential as promising VGCC α2δ-1 subunit inhibitor.

On the other hand, dencandrol F exhibits a distinct binding pattern, forming hydrogen bonds with Glu665 and Asp665, while engaging in hydrophobic interactions with residues like Lys498, Arg499, Thr501, Arg503, Phe504, Tyr512, Leu523, Pro525, and His524 (Table 2 & Figure 1). This unique set of interactions suggests that CMNPD31516 may bind to a different region of the α2δ-1 subunit, might be modulating its activity through an alternative mechanism. Whereas CMNPD23679 (ent-1(10)-aristolen-9β-ol) did not form any direct hydrogen bonds with the target protein. However, it established hydrophobic contacts with residues such as Asn509, Tyr511, Glu565, His524, Pro525, Lys593, Asn526, and Lys765 (Table 2 & Figure 1). These interactions highlight the importance of hydrophobic forces in stabilizing ligand-protein complexes, even in the absence of direct hydrogen bonding. Marine phytochemical acanthiline-A is an evergreen spinus herb traditionally used in Chinese medicine against rheumatism, paralysis, asthma, and antiinflammatory, anti-hepatitis agent. According to the prior studies, its alcoholic extract showed antioxidant, hepatoprotective, antitumor, and anticarcinogenic effects [15].

Ceriops decandra, a mangrove plant, exhibits diverse therapeutic activities as supported by its traditional medicinal uses and phytochemical studies. Different plant parts are utilized for treating diarrhea, dysentery, skin diseases, ulcers, rheumatism, and inflammatory conditions [16,17]. Extracts and isolated compounds from C. decandra have demonstrated antioxidant, antimicrobial, anti-inflammatory, and cytotoxic activities [17,18], validating its ethnomedicinal applications. The red alga Laurencia similis has been extensively studied for its rich repertoire of bioactive secondary metabolites. Kamada and Vairappan (2013) investigated a Bornean population of L. similis and identified seven compounds, including ent-1(10)-aristolen-9β-ol (1), a new optical isomer of 1(10)-aristolen-9-ol [19]. Notably, compounds 1, 4 (9-aristolen-1α-ol), and 5 (2,3,5,6-tetrabromoindole) exhibited potent antibacterial activity against antibiotic-resistant clinical bacterial strains and cytotoxic effects against selected cancer cell lines, highlighting the therapeutic potential of the metabolites from this alga. These plant extracts previously showed therapeutic activity, and docking studies of these compounds showed better binding affinity against the epileptic target "VGCC sub-chain α2δ". Hence, these compounds can be a promising candidate as anti-epileptic drugs, which can be further investigated in-vivo studies. In the present study, these phytochemicals were found to exhibit better binding affinities towards the α2δ-1 subunit of VGCCs compared to the standard anticonvulsant drugs, gabapentin and pregabalin. This suggests that these natural compounds may possess potential antiepileptic properties by modulating the activity of VGCCs, particularly the α2δ-1 subunit, leading to the inhibition of calcium influx into neurons.

## Conclusion:

Numerous anti-epileptic medications are helpful in the early or middle stages of the disease and do not effectively treat it. Hence, we report that terrestrial ascaridole have highest binding affinity (-7.3 kcal/mol) for further in-vitro, and in vivo validation studies.

## Figures and Tables

**Table 1:** The phytochemicals showing better binding affinities (lower binding energies) than the standard (Pregabilin, when docked against α2δ-1 subunit of VGCC protein

**CMNPD_ID**	**PubChem-ID**	**Binding energy (Kcal/Mol)**
CMNPD31502	163113318	-11.9
CMNPD31516	163112924	-10.9
CMNPD23679	162850358	-9
CMNPD29266	162848167	-8.9
CMNPD25048	163109360	-8.7
CMNPD766	23425277	-8.3
CMNPD2640	101957588	-8.3
CMNPD13364	11143863	-8
CMNPD858	11020496	-7.8
CMNPD11779	10966444	-7.8
CMNPD729	163056147	-7.7
CMNPD13363	10938244	-7.7
CMNPD703	23426954	-7.6
CMNPD702	23426953	-7.6
CMNPD95	163056145	-7.5
CMNPD5977	101838115	-7.5
CMNPD2890	162924843	-7.5
CMNPD12541	637399	-7.5
CMNPD718	23425290	-7.3
CMNPD705	23426956	-7.3
CMNPD4220	14565461	-7.3
CMNPD25047	102233553	-7.3
CMNPD14904	21778494	-7.3
CMNPD4791	163042085	-7.2
CMNPD31550	162860522	-7.2
CMNPD19981	46197377	-7.2
CMNPD11787	21776056	-7.2
CMNPD728	163078685	-7.1
CMNPD727	14565942	-7.1
CMNPD717	162911090	-7.1
CMNPD5972	163056142	-7.1
CMNPD2261	163056146	-7.1
CMNPD730	162849118	-7
CMNPD4208	163068624	-7
CMNPD27849	132525079	-7
CMNPD15845	21778794	-7
CMNPD6621	10019803	-6.9
CMNPD4196	14729454	-6.9
CMNPD3713	21638192	-6.9
CMNPD31514	163118934	-6.9
CMNPD27851	132525080	-6.9
CMNPD20000	162906510	-6.9
CMNPD1907	3010316	-6.9
CMNPD1857	14355384	-6.9
CMNPD11780	10969077	-6.9
Pregabalin	5486971	-6.8

**Table 2:** H-bond and hydrophobic interactions of selected phytochemicals when docked against VGCC α2δ-1 protein

**CMNPD_ID**	**Hydrogen bonds**	**Hydrophobic interactions**
CMNPD31502	Gln 1033, Phe780	Arg598, Tyr779, Lys782, Phe858,Gly872, Arg873, Phe874, Glu1035,
CMNPD31516	Glu665, Asp 665	Lys498, Arg499,Thr501, Arg503, Phe504, Tyr512, Leu523, Pro525, His524,
CMNPD23679		Asn509,Tyr511, Glu565, His524, Pro525, Lys593, Asn526, Lys765,
Pregabalin	Gln1033, Arg598	Tyr779, Phe780 Phe874, Arg873, Gly872, Phe858, Ala1034
